# Prognostic Value of Biomarkers in Acute Aortic Dissection: Analysis of Clinical Outcomes and Mortality

**DOI:** 10.1155/emmi/6664490

**Published:** 2025-03-18

**Authors:** Ömer Faruk Turan, Nurullah İshak Işık, Safa Dönmez, Hamdi Haluk Çalı, Kasım Ateş, Feyza Baysar, Lukasz Szarpak, Jacek Smereka, Burak Katipoğlu

**Affiliations:** ^1^Emergency Medicine Department, Ankara Etlik City Hospital, Ankara, Türkiye; ^2^Emergency Medicine Attending, General Directorate of Health Services, Republic of Turkey Ministry of Health, Ankara, Türkiye; ^3^Emergency Medicine Department, Health Sciences University Ankara City Hospital Health Practice and Research Center, Ankara, Türkiye; ^4^Emergency Medicine Department, Yozgat City Hospital, Yozgat, Türkiye; ^5^Department of Clinical Research and Development, LUXMED Group, Warsaw, Poland; ^6^Institute of Medical Science, Collegium Medicum, The John Paul II Catholic University of Lublin, Lubin, Poland; ^7^Henry JN Taub Department of Emergency Medicine, Baylor College of Medicine, Houston, Texas, USA; ^8^Department of Emergency Medical Service, Wroclaw Medical University, Wroclaw, Poland

**Keywords:** acute aortic dissection, biomarkers, emergency services, mortality prediction

## Abstract

**Introduction:** Acute aortic dissection (AAD) is a severe condition requiring immediate diagnosis and treatment to reduce high mortality rates. This study investigates laboratory markers that may support the diagnostic process and predict surgical outcomes and mortality in AAD patients.

**Materials and Methods:** This retrospective study analyzed data from 85 patients diagnosed with AAD in an emergency setting. Patients over 18 years of age with a diagnosis of acute dissection were included. Key laboratory and clinical parameters were examined to determine their association with mortality and the likelihood of surgical intervention.

**Results:** The study found that younger patients were more likely to undergo surgery, while parameters such as white blood cells (WBCs), neutrophil, and lymphocyte counts were elevated in those undergoing surgery. Mortality predictors included elevated mean platelet volume (MPV), low pH, bicarbonate (HCO_3_), and base deficit levels. Each unit increase in MPV was associated with a threefold increase in mortality risk, and DeBakey Type 1 patients exhibited the highest MPV levels.

**Discussion:** WBC and MPV values were linked with surgical and mortality outcomes, respectively. Blood gas analysis parameters, particularly HCO_3_ and base deficit levels, were significant mortality predictors, underscoring the importance of metabolic markers in AAD assessment. The findings suggest that incorporating these laboratory parameters into diagnostic and treatment decisions could improve AAD management.

## 1. Introduction

Emergency departments are dynamic, high-stress environments characterized by rapid patient turnover. Within these settings, providing accurate diagnosis, timely intervention, and appropriate patient referral within a constrained timeframe is essential. Prompt identification and management of life-threatening conditions, such as acute myocardial infarction, pulmonary thromboembolism, and acute aortic dissection (AAD), are critical to reducing morbidity and mortality. The capacity to diagnose and address these conditions within minutes or hours is paramount [1].

AAD is a rare but high-risk condition associated with considerable morbidity and mortality, characterized by separation or rupture between vessel wall layers [1]. The incidence of AAD is estimated to be between 2.6 and 3.5 per 100,000 individuals [2, 3]. The diagnosis of AAD presents significant challenges, with high rates of misdiagnosis [4]. Studies indicate that approximately one-third of AAD patients are misdiagnosed at initial presentation, and one-quarter receive a correct diagnosis only after 24 h [5]. Delays in appropriate treatment markedly increase morbidity and mortality [6]. Despite accurate diagnosis, in-hospital mortality remains as high as 27% [7, 8].

Depending on its anatomical location, AAD may present with diverse signs and symptoms [9]. Although the symptomatology is broad, common presentations include back pain, chest pain, and syncope, often described as a tearing sensation [10, 11]. The broad spectrum of presenting complaints contributes to diagnostic difficulty and delays in intervention [12]. This underscores the challenges emergency physicians face, who are often the first point of contact for patients with AAD. The nonspecific nature of symptoms and the absence of clear diagnostic criteria exacerbate diagnostic uncertainty [13]. Consequently, clinicians have sought biomarkers to facilitate accurate diagnosis through blood parameters upon presentation.

Various biomarkers, including inflammatory indicators, vascular wall proteins, and coagulation factors, have been investigated for their diagnostic potential in AAD [14, 15]. For example, mean platelet volume (MPV), a parameter easily obtained from routine emergency department hemogram tests, has been studied due to its accessibility, speed, and cost-effectiveness [16]. However, no biomarker has yet demonstrated sufficient sensitivity and specificity to be widely adopted, and thus, there remains no universally accepted biomarker for AAD diagnosis [17]. Although some supportive diagnostic tools are available, computed tomographic angiography (CTA) remains the gold standard for confirming AAD [12].

This study evaluates cases of AAD diagnosed in the emergency department, aiming to identify early indicators that could support rapid and accurate diagnosis in this challenging setting. Additionally, we investigated parameters that may predict mortality, thereby assisting clinicians in managing cases with high clinical suspicion of AAD. We aimed to derive values from blood parameters at admission that may inform clinical scoring systems for future diagnostic support.

## 2. Materials and Methods

This study is a retrospective observational analysis conducted between October 1, 2022, and April 30, 2024. The sample included patients diagnosed with AAD through thoracoabdominal CTA at the Etlik City Hospital Adult Emergency Department. Patients were assessed according to predetermined inclusion and exclusion criteria, with 85 cases meeting the eligibility requirements. Ethics committee approval was granted by the Etlik City Hospital Ethics Committee (decision number AEŞH-BADEK-2024-852).

### 2.1. Inclusion Criteria

• Age 18 years or older
• Diagnosis of AAD in the emergency department

### 2.2. Exclusion Criteria

• Imaging performed due to trauma
• Patients referred from external centers with a prior AAD diagnosis undergoing repeated imaging
• Patients with a prior diagnosis of chronic aortic dissection
• Patients younger than 18 years
• Patients with missing data

Surgical approaches were classified as cardiothoracic/vascular surgery or endovascular stent-graft placement, depending on the patient's clinical presentation, anatomical classification, and institutional protocols [18]. In-hospital mortality was considered as the primary mortality endpoint.

Patients were categorized based on the DeBakey Classification of Aortic Dissection [8, 18], which differentiates AAD cases according to their anatomical involvement.

• DeBakey Type I: This type originates in the ascending aorta and extends beyond the aortic arch, often involving the descending thoracic and abdominal aorta. It is the most extensive and carries the highest risk of complications.
• DeBakey Type II: This type originates in and is confined to the ascending aorta without extension beyond the arch. Due to the risk of rupture, tamponade, and aortic valve involvement, it often necessitates urgent surgical intervention.
• DeBakey Type III: Originates in the descending aorta (distal to the left subclavian artery).

**Figure 1:**
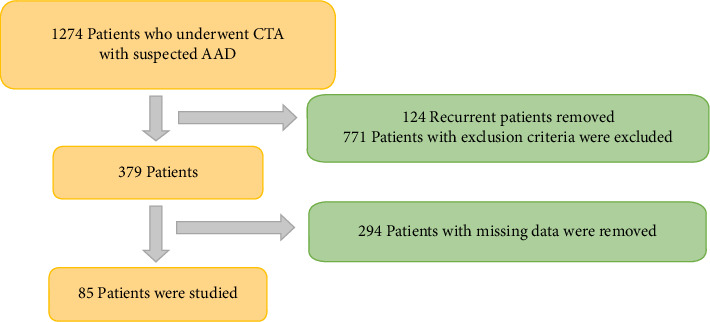
Flowchart.

The data pool included patients who underwent thoracoabdominal CTA for suspected AAD. Of the initial cohort of 1274 patients, 771 were excluded due to alternative diagnoses. Additionally, 124 patients were removed due to duplicate entries from repeated imaging and test orders. Laboratory results or follow-up values were unavailable for 294 patients, as some laboratory parameters were not recorded, and no further medical records were available after CT imaging for a subset of patients. After these exclusions, the final study cohort comprised 85 patients.  Figure 1 provides a detailed flowchart illustrating the patient selection process and exclusion criteria.

### 2.3. Statistical Analysis

Data analysis was conducted using SPSS Version 28 (IBM Corp., Armonk, NY, USA). The normality of data distribution was assessed through visual inspections of histograms and *Q–Q* plots, as well as statistical tests, including the Shapiro–Wilk test and Kolmogorov–Smirnov test. Visual inspections via histograms and *Q–Q* plots provided insights into data distribution, while the Shapiro–Wilk test was mainly applied to assess normality for small sample groups. The Kolmogorov–Smirnov test was used to test the general conformity of data distribution in larger samples. Depending on whether data met the normality assumption, parametric or nonparametric tests were employed for analysis. Intergroup comparisons of demographic and clinical parameters utilized the independent samples *t*-test for normally distributed continuous variables. At the same time, the Mann–Whitney *U* test was applied for non-normally distributed data. The Pearson chi-square test was used for categorical variables. For multivariate analyses, multiple logistic regression was applied to identify independent variables influencing mortality and the need for surgery. Variables with a *p* value below 0.2 in binary analysis were included in the model, and the final model was determined via backward elimination. Results are reported with 95% confidence intervals and *p* values, with *p* < 0.05 considered statistically significant.

## 3. Results

The analysis included 85 patients diagnosed with AAD in the emergency department, comprising 51 male and 34 female patients, with 23 patients experiencing mortality. Pairwise comparisons based on mortality status are presented in Table 1. No significant differences were observed between the mortality and survivor groups regarding age (*p*=0.730) or gender (*p*=0.457). Levels of white blood cells (WBCs), neutrophils, lymphocytes, and platelets were similar across groups (*p* > 0.05). However, MPV was significantly higher in the mortality group (*p*=0.033), while pH (*p*=0.017), bicarbonate (HCO_3_) (*p*=0.004), and base deficit (*p* < 0.001) were significantly lower. The mortality group also exhibited higher urea levels (*p*=0.024), though the difference in creatinine levels was not statistically significant (*p*=0.103). Glomerular filtration rate (GFR) was lower in the mortality group (*p*=0.016), and troponin levels were significantly elevated (*p*=0.046). Furthermore, DeBakey Type 1 dissection was more prevalent in the mortality group (*p*=0.014).

A multiple logistic regression analysis was conducted to determine independent variables associated with mortality (Table 2). Parameters with *p* values below 0.2 in pairwise comparisons were included in the regression model, which was finalized using backward elimination. The optimized model at step 6 revealed that elevated MPV was significantly associated with increased mortality, with each unit increase in MPV raising mortality risk by approximately threefold (Exp (B) = 3.062, 95% CI: 1.35–6.90, *p*=0.007). HCO_3_ levels were inversely related to mortality, with each unit increase decreasing mortality risk by 19% (Exp (B) = 0.810, 95% CI: 0.68–0.96, *p*=0.019). Creatinine levels demonstrated a positive association with mortality, with each unit increase correlating with a 47% increase in mortality risk (Exp (B) = 1.470, 95% CI: 1.00–2.14, *p*=0.045). In addition, an analysis based on the DeBakey classification revealed that Type 2 dissections were associated with a lower mortality risk compared to Type 1 (OR = 0.329, *p*=0.297); however, this finding was not statistically significant. In contrast, Type 3 dissections were significantly associated with a markedly lower mortality risk than Type 1 (OR = 0.057, *p*=0.011, *p* < 0.05).

Table 3 presents pairwise comparisons based on surgical intervention status. Differences in age, WBC, neutrophil, and lymphocyte levels were significant between patients who underwent surgery and those who did not. Patients who underwent surgery were younger (*p* < 0.001), had higher WBC (*p* < 0.001) and neutrophil (*p*=0.033) levels, and exhibited elevated lymphocyte levels (*p* < 0.001). No significant differences were observed in platelet count, MPV, pH, HCO_3_, or base deficit. Platelet-to-lymphocyte ratio (PLR) (*p*=0.025) and urea levels (*p*=0.025) were significantly lower in the surgical group. Differences in troponin and *D*-dimer levels were not statistically significant (*p* > 0.05).

In the multiple logistic regression analysis examining variables influencing surgical intervention (Table 4), parameters with *p* values below 0.2 in pairwise comparisons were included, and the final model was optimized at step 3 using backward elimination. Results indicated that age was negatively associated with the likelihood of surgery, with each unit increase in age reducing the probability of surgery by 5% (Exp (B) = 0.946, 95% CI: 0.907–0.986, *p*=0.008). Neutrophil levels were positively associated with the probability of surgery, with each unit increasing the likelihood of surgery by 18% (Exp (B) = 1.181, 95% CI: 1.019–1.369, *p*=0.027). Similarly, each unit increase in lymphocyte level doubled the probability of surgical intervention (Exp (B) = 1.969, 95% CI: 1.141–3.396, *p*=0.015). Each unit increase in urea level was associated with a 6% increase in the probability of surgery (Exp (B) = 0.940, 95% CI: 0.893–0.990, *p*=0.020). No significant association was observed between GFR and surgical intervention (*p*=0.116).

## 4. Discussion

AAD is a challenging condition to diagnose, requiring extensive diagnostic effort and presenting a high mortality risk [3, 13]. This study evaluated laboratory parameters that may facilitate the diagnostic process and predictors of surgical outcomes and mortality.

Our results showed a mean patient age of 62, consistent with previous studies [4, 5, 8]. We observed that younger patients were statistically more likely to undergo surgery, potentially due to lower vascular fragility and fewer comorbid conditions. Although we did not find a statistically significant relationship between age and mortality, the fact that younger patients underwent surgery more frequently suggests that advanced age may be a critical factor. Age and associated comorbidities may incline clinicians toward nonsurgical management without increasing in-hospital mortality.

WBC, neutrophil, and lymphocyte counts were significantly elevated in patients who proceeded to surgery. AAD is expected to increase as acute phase reactants like WBC, neutrophil, and lymphocyte counts rise secondary to vessel wall pathologies [11]. However, we did not observe a significant elevation in WBC levels among patients with fatal outcomes. Supporting our findings, Suzuki et al. and Feng et al. also reported no association between WBC elevation and mortality [19, 20]. Dongze et al., in contrast, found WBC elevation to be significant for long-term mortality, indicating that while WBC may not predict immediate mortality, it may serve as a long-term prognostic indicator [16]. Overall, while mortality prediction is a common focus, studies on surgical prediction in AAD are rare. Our findings highlight the potential value of elevated WBC, neutrophil, and lymphocyte levels in predicting the need for surgical intervention, contributing an essential perspective to the literature.

Proinflammatory cytokines and acute phase reactants in inflammatory processes affect megakaryocytopoiesis, decreasing platelet count and volume. Low platelet count is a poor prognostic indicator in many conditions [21, 22], and trends in platelet counts are essential for patient monitoring. In AAD, a condition with sudden onset and high mortality risk, patients may deteriorate before a decrease in platelets is observed [21–23]. MPV, a more reliable marker of platelet activity than platelet count, is commonly used as a mortality predictor across conditions, including liver and kidney dysfunction and cardiovascular bypass surgery [24–26]. Ge et al. and Zhang et al. found higher MPV values among AAD patients with fatal outcomes [27, 28]. In our study, elevated MPV was a statistically significant predictor of mortality, with each unit increasing the mortality risk threefold. Additionally, we found the highest MPV levels in our DeBakey Type 1 patient—the subtype with the highest mortality rate—further supporting the potential role of MPV as a prognostic indicator in more extensive population studies.

Blood gas analysis is critical in assessing acute myocardial infarction, pulmonary thromboembolism, cerebrovascular disease, sepsis, and other life-threatening conditions with rapid progression [29–31]. In the emergency department, blood gas analysis is frequently used for patients presenting with severe pain, instability, or a high mortality risk [32]. Our study found that HCO_3_, base deficit, and pH levels were statistically significant predictors of mortality, with worse blood gas values correlating with poorer outcomes. We found that each unit increase in HCO_3_ decreased mortality risk by 19%, indicating that patients capable of counteracting metabolic disturbances may have better outcomes. Our findings align with those of Tan et al., who reported a similar relationship between HCO_3_ levels and mortality in AAD [33]. Our study also observed that blood gas values among surgical patients were near-normal, suggesting that while abnormal blood gas values may support an AAD diagnosis, normal values should not be used to rule out the condition.

Beyond laboratory markers, surgical decision making in AAD is primarily guided by disease severity, anatomical classification, hemodynamic stability, and imaging findings [18]. The DeBakey classification, which serves as the foundation of our study, is a key determinant in selecting surgical candidates. However, hemodynamic stability remains a critical factor in clinical decision making. In our cohort, 45% of patients underwent surgical intervention, a rate comparable to the 40% reported by Yeh, Su, and Liu across all age groups and dissection subtypes [18, 34]. Similarly, Melvinsdottir et al. reported a 55% surgical intervention rate among patients with Type 1 and Type 2 dissections [2]. While our findings are proportionally consistent with the literature, no statistically significant differences were observed between dissection subtypes. This lack of significance may be attributed to the study's limited sample size, which could have restricted the ability to detect meaningful statistical differences.

Our study's in-hospital mortality rate was 26%, aligning with the figures reported in other studies [8]. When out-of-hospital fatalities are included, this rate climbs significantly. In a 21 year retrospective study of Icelandic hospital records, Melvinsdottir and colleagues reported a combined in- and out-of-hospital mortality rate of 39% and a 30-day mortality rate of 54.9% for AAD patients [2]. Similarly, Arturo Evangelista and the International Association of Acute Aortic Dissection found that mortality in AAD patients ranged from 36% to 72% within the first 48 h after admission to the ICU, based on 20 years of data [35]. When we further analyzed mortality rates based on dissection subtypes, we found that, consistent with the literature, mortality was significantly higher in Type 1 dissections and lower in Type 3 dissections [8, 18]. These high rates underscore the critical importance of timely diagnosis and intervention. The findings in our study emphasize the role of early diagnostic markers and reveal a continued need for research to identify predictive parameters.

While our findings illustrate the value of specific blood parameters in diagnosing and predicting AAD outcomes, we believe that laboratory tests alone may not be sufficient for comprehensive assessment. Long and colleagues similarly advocated integrating AAD-specific scoring systems in clinical decision making [36]. Combining laboratory data with structured scoring systems could improve early diagnostic accuracy and treatment planning for this highly fatal condition.

In our study, patients who underwent surgery and those who experienced mortality were evaluated as separate groups based on biochemical markers. Elevated WBC, neutrophil, and lymphocyte counts, as well as higher PLR, were associated with surgical intervention, suggesting that a heightened inflammatory response may play a role in surgical candidacy. In contrast, elevated MPV, troponin, and abnormal blood gas parameters (including reduced pH, bicarbonate, and base deficit) were significant predictors of mortality. These findings reinforce the clinical utility of inflammatory and metabolic markers in both risk stratification and surgical decision making.

Additionally, although *D*-dimer is widely recognized as a valuable diagnostic marker for AAD, our results indicate that it lacks prognostic utility in patient follow-up and mortality prediction. This highlights the need for further research into alternative biomarkers that may provide more accurate prognostic insights. Future studies with larger, multicenter cohorts should focus on validating these findings and investigating novel laboratory markers that could improve early risk assessment and guide treatment decisions.

## 5. Limitations

A limitation of our study was the exclusion of certain patients due to missing data, which may have influenced our findings. Additionally, including only emergency department admissions with AAD limits the generalizability of our results. Moreover, the retrospective, single-center design may affect external validity. Larger, multicenter prospective studies are needed to confirm our findings.

## Figures and Tables

**Table 1:** Association of demographic and clinical parameters with mortality.

Parameters		Groups (mortality)		95% CI	*p* value
		Survival	Nonsurvivals		
Age, mean ± SD		62.4 ± 13.5	61.2 ± 18.3	−8.54–6.01	0.730⁣^∗^

Gender, *n* (%)	Male	39 (45.9)	12 (14.1)		0.457^†^
	Female	23 (27.1)	11 (12.9)		

WBC, mean ± SD		10.78 ± 4.23	10.47 ± 3.33	−2.25–1.64	0.755⁣^∗^

Neu, mean ± SD		7.83 ± 4.14	7.79 ± 3.80	−2.01–1.93	0.969⁣^∗^

Lymph, mean ± SD		2.09 ± 1.44	2.04 ± 1.39	−0.74–0.64	0.894⁣^∗^

Plt, mean ± SD		250.41 ± 118.98	213.30 ± 104.98	−93.17–18.94	0.192⁣^∗^

NLR, median (25%–75%)		3.96 (2.26–7.54)	5.16 (1.30–9.11)		0.805^‡^

PLR, median (25%–75%)		132.50 (76.49–238.66)	134.00 (63.22–260.60)		0.610^‡^

MPV, mean ± SD		10.24 ± 0.87	10.74 ± 1.14	0.04–0.96	0.033⁣^∗^

pH, mean ± SD		7.39 ± 0.06	7.33 ± 0.10	−0.10–−0.01	0.017⁣^∗^

HCO_3_, mean ± SD		24.24 ± 4.79	20.93 ± 4.46	−5.65–−1.14	0.004⁣^∗^

Lactate, median (25%–75%)		1.70 (1.21–2.40)	2.00 (1.10–4.90)		0.372^‡^

Base deficit, mean ± SD		−0.82 ± 4.37	−4.56 ± 5.12	−6.03–−1.60	< 0.001⁣^∗^

Urea, mean ± SD		42.27 ± 18.43	59.92 ± 35.55	2.63–33.69	0.024⁣^∗^

Creatinine, median (25%–75%)		0.90 (0.80–1.30)	1.22 (0.86–7.86)		0.103^‡^

GFR, mean ± SD		75.00 ± 29.46	44.70 ± 36.00	−32.55–−3.67	0.016⁣^∗^

Troponin, median (25%–75%)		8.90 (4.05–15.00)	49.80 (17.57–94.07)		0.046^‡^

D-dimer, median (25%–75%)		4.10 (1.75–9.20)	10.65 (2.31–22.15)		0.104^‡^

DeBakey, *n* (%)	1	30 (35.3)	19 (22.4)		0.014^†^
	2	8 (9.4)	2 (2.4)		
	3	24 (28.2)	2 (2.4)		

⁣^∗^Independent samples *t*-test; mean ± standard deviation.

^†^Pearson chi-squared test; *n* (%).

^‡^Mann–Whitney *U* test; median (25%–75% quartiles).

**Table 2:** Multiple logistic regression analysis: independent variables affecting mortality.

		B	*p* value	Exp (B)	95% CI for EXP (B)
Step 6^a^	MPV	1.119	0.007	3.062	1.35–6.90
	HCO_3_	−0.210	0.019	0.810	0.68–0.96
	Creatinine	0.385	0.045	1.470	1.00–2.14
	Classification⁣^∗^		0.032		
	DeBakey Type 2	−1.113	0.297	0.329	0.04–2.66
	DeBakey Type 3	−2.866	0.011	0.057	0.06–0.52
	Constant	−9.420	0.022	0.000	

*Note:* Variable(s) entered on step 1: PLT, MPV, pH, HCO_3_, base deficit, urea, creatine, GFR, and classification.

⁣^∗^In the logistic regression analysis, DeBakey Type 1 was used as the reference category. The coefficients for DeBakey Type 2 and DeBakey Type 3 were calculated in comparison to this reference category.

^a^0.05.

**Table 3:** Associations of demographic and clinical parameters affecting surgery.

Parameters		Groups (surgery)		95% CI-diff	*p* value
		Nonsurgical	Surgical		
Age, mean ± SD		67.6 ± 15.3	57.4 ± 12.9	4.11–16.30	< 0.001⁣^∗^

Gender, *n* (%)	Male	22 (25.9)	29 (34.1)		0.534^†^
	Female	17 (20.0)	17 (20.0)		

WBC, mean ± SD		9.16 ± 3.54	12.00 ± 3.93	−4.46–−1.21	< 0.001⁣^∗^

Neu, mean ± SD		6.81 ± 3.59	8.68 ± 4.22	−3.57–−0.15	0.033⁣^∗^

Lymph, mean ± SD		1.55 ± 0.82	2.52 ± 1.66	−1.53–−0.41	< 0.001⁣^∗^

Plt, mean ± SD		252.91 ± 118.95	237.84 ± 109.88	−44.97–55.99	0.829⁣^∗^

NLR, median (25%–75%)		4.36 (2.15–9.33)	3.86 (2.22–7.26)		0.549^‡^

PLR, median (25%–75%)		169.16 (85.94–268.88)	110.57 (62.73–169.69)		0.025^‡^

MPV, mean ± SD		10.46 ± 1.17	10.31 ± 0.78	−0.30–0.58	0.529⁣^∗^

pH, median (25%–75%)		7.37 (7.33–7.42)	7.39 (7.36–7.42)		0.331⁣^∗^

HCO_3_, median (25%–75%)		24.35 (20.92–25.37)	23.50 (20.87–25.00)		0.200⁣^∗^

Lactate, median (25%–75%)		1.68 (1.30–2.38)	1.70 (1.20–3.10)		0.777^‡^

Base deficit, mean ± SD		−1.61 ± 5.49	−1.90 ± 4.35	−1.86–2.37	0.811⁣^∗^

Urea, median (25%–75%)		47.00 (33.40–66.50)	36.00 (28.47–53.32)		0.025^‡^

Creatine, median (25%–75%)		1.00 (0.80–1.32)	1.00 (0.80–1.30)		0.842^‡^

GFR, mean ± SD		63.10 ± 27.94	72.06 ± 27.09	−20.16–3.82	0.179⁣^∗^

Troponin, median (25%–75%)		31.00 (6.00–78.10)	9.70 (4.67–15.20)		0.055^‡^

D-dimer, median (25%–75%)		2.00 (1.75–22.00)	7.50 (2.86–12.22)		0.334^‡^

DeBakey, *n* (%)	Type 1	21 (24.7)	28 (32.9)		0.611^†^
	Type 2	4 (4.7)	6 (7.1)		
	Type 3	14 (16.5)	12 (14.1)		

⁣^∗^Independent samples *t*-test; mean ± standard deviation.

^†^Pearson chi-squared test; *n* (%).

^‡^Mann–Whitney *U* test; median (25%–75% quartiles).

**Table 4:** Multiple logistic regression analysis: independent variables affecting surgical procedure.

		B	*p* value	Exp (B)	95% CI for EXP (B)
Step 3^a^	Age	−0.056	0.008	0.946	0.907–0.986
	Neutrophil	0.167	0.027	1.181	1.019–1.369
	Lymphocyte	0.677	0.015	1.969	1.141–3.396
	Urea	−0.061	0.020	0.940	0.893–0.990
	GFR	−0.028	0.116	0.972	0.938–1.007
	Constant	5.754	0.076	315.540	

^a^Variable(s) entered on step 1: age, WBC, neutrophile, lenfosite, PLR, urea, and GFR.

## Data Availability

All data generated or analyzed during this study are included in this published article. Data and materials are accessible and can be shared when necessary.
